# Relative Bradycardia in Patients with Mild-to-Moderate Coronavirus Disease, Japan

**DOI:** 10.3201/eid2610.202648

**Published:** 2020-10

**Authors:** Kazuhiko Ikeuchi, Makoto Saito, Shinya Yamamoto, Hiroyuki Nagai, Eisuke Adachi

**Affiliations:** The University of Tokyo, Tokyo, Japan

**Keywords:** relative bradycardia, bradycardia, coronavirus disease, COVID-19, mild-to-moderate coronavirus disease, severe acute respiratory syndrome coronavirus 2, SARS-CoV-2, coronaviruses, viruses, respiratory infections, Japan

## Abstract

Coronavirus disease is reported to affect the cardiovascular system. We showed that relative bradycardia was a common characteristic for 54 patients with PCR-confirmed mild-to-moderate coronavirus disease in Japan. This clinical sign could help clinicians to diagnose this disease.

Pulse rate usually increases 18 beats/min for each 1°C increase in body temperature (*1*). However, in some specific infectious diseases, pulse rate does not increase as expected, a condition called relative bradycardia. High fever (temperature >39°C) for patients with coronavirus disease (COVID-19) has been reported (*2*,*3*), but the association between fever and pulse rate has not been investigated. We investigated relative bradycardia as a characteristic clinical feature in patients with mild-to-moderate COVID-19.

Retrospective analyses of routinely collected clinical records of COVID-19 patients were approved by the ethics committee of the Institute of Medical Science, The University of Tokyo (approval no. 2020–5-0420). During March 1–May 14, we identified all adult hospitalized patients with COVID-19 at a university hospital in Tokyo, Japan. We confirmed diagnoses of COVID-19 by using reverse transcription PCR. Patients who had known factors that could affect pulse rate (e.g., concurrent conditions or medications) were excluded.

We obtained the highest body temperature in each day during hospitalization and the pulse rate at the time. To account for within-person correlation, we used 2-level mixed-effects linear regression (with random intercept) for analysis of factors associated with pulse rate: age, sex, time from first symptoms, systolic blood pressure, diastolic blood pressure, respiratory rate, and percutaneous oxygen saturation.

We performed variable selection by backward elimination using a p value of 0.05 by likelihood ratio test as the cutoff value. We performed statistical analysis by using Stata MP 15.1 (StataCorp, https://www.stata.com). Relative bradycardia was defined as an increase in pulse rate <18 beats/min for each 1°C increase in body temperature (*1*).

During the study period, 57 patients with COVID-19 were admitted to our hospital (Table); 3 patients were excluded (2 were receiving beta-blockers and 1 had a pulmonary embolism). The median age was 45.5 years (range 20–81 years), and 72.2% (39/54) of patients were male. Median time from the appearance of first symptoms to admission was 9 days (range 2–25 days). At admission, median body temperature was 37.2°C (range 36.1°C–39.2°C), pulse rate 84 beats/min (range 62–134 beats/min), and systolic blood pressure, 116 mm Hg (range 80–170 mm Hg). During admission, 13.0% (7/54) of patients had high fever (temperature >38.9°C), and all had a pulse rate <120 beats/min (range 72–114 beats/min).

**Table:** Characteristics of patients with relative bradycardia and mild-to-moderate coronavirus disease, Japan

Characteristic	No. assessed	Value*
Age, y	54	45.5 (20–81)
Sex		
M	39	39 (72.2)
F	15	15 (27.8)
Body mass index, kg/m^2^	54	23.7 (15.9–51.1)
Current smoker	48	16 (33.3)
Days from symptom onset to admission	54	9 (2–25)
Vital signs at admission		
Body temperature, °C	54	37.2 (36.1–39.2)
Pulse rate, beats/min	54	84 (62–134)
Systolic blood pressure, mm Hg	54	116 (80–170)
Diastolic blood pressure, mm Hg	54	70.5 (51–124)
Respiratory rate, breaths/min	53	18 (16–26)
Percutaneous oxygen saturation, %†	54	97 (92–100)
Highest temperature during admission,°C	54	
<37.5	54	27 (50.0)
37.5–38.9	54	20 (37.0)
>38.9°C	54	7 (13.0)
Laboratory findings at admission		
Leukocyte count, cells/mm^3^	54	5,530 (2,690–16,700)
Lymphocyte count, cells/mm^3^	54	1,251 (381–2,852)
Hemoglobin, g/dL	54	14.7 (11.1–17.3)
Platelet count, × 1,000/mm^3^	54	231 (106–444)
Blood urea nitrogen, mmol/L	54	4.3 (2.1–7.9)
Creatinine, μmol/L	54	69.0 (34.5–120.2)
Sodium, mmol/L	53	139 (132–148)
Potassium, mmol/L	53	4.0 (3.1–4.8)
Creatine kinase, U/L	52	74 (22–674)
C-reactive protein, mg/L	54	17.9 (0.1–215.6)
Brain natriuretic peptide, pg/mL	52	5.8 (5.8–43.2)
D-dimer, mg/L	50	0.5 (0.5–6.5)
Concurrent conditions		
Hypertension	54	8 (14.8)
Diabetes	54	5 (9.3)
Chronic obstructive pulmonary disease	54	1 (1.9)
Coronary heart disease	54	0 (0)
HIV Infection	54	4 (7.4)

*Values are median (range) or no. (%). †Nine patients required oxygen therapy at admission.

We performed computed tomography and electrocardiography for all patients: no patients were given a diagnosis of cardiac disease. Computed tomography showed pneumonia for 49 (90.7%) patients, and 11 (20.4%) patients required oxygen therapy without intubation. A total of 24 patients received COVID-19–specific treatment (favipiravir, n = 15; hydroxychloroquine, n = 10; both drugs, n = 1); no patients received vasopressors, or corticosteroids for COVID-19. All patients improved and were discharged.

Body temperature, respiratory rate, systolic blood pressure, and time after the first symptoms (in days) were associated with pulse rate by univariable analysis (Appendix Table). However, only body temperature was independently associated with pulse rate by multivariable analysis. The predicted change in pulse rate was 7.37 (95% CI 5.92–8.82) beats/min for each 1°C increase in body temperature (Figure).

**Figure:**
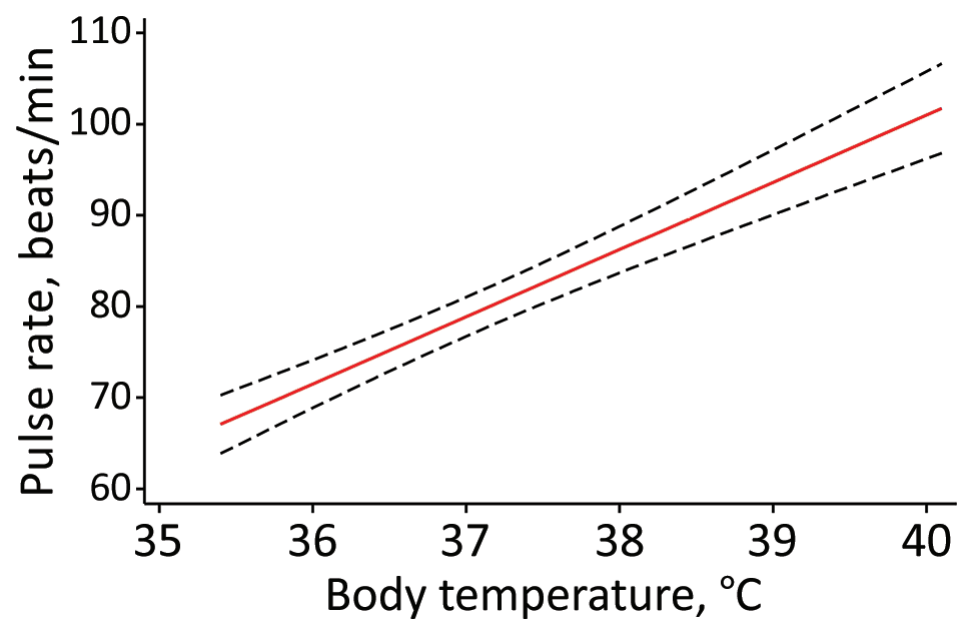
Predicted pulse rate over body temperature (red line) based on final random intercept model for relative bradycardia in patients with mild-to-moderate coronavirus disease, Japan. Black dashed lines indicate 95% CIs.

Relative bradycardia is a characteristic physical finding in some intracellular bacterial infections, viral infections, and noninfectious diseases (*4*). Our data showed that a predicted change in pulse rate was <18 beats/min for each 1°C increase in patients with COVID-19. Furthermore, all patients with high fever also met another criterion of relative bradycardia (i.e., body temperature >38.9°C with pulse rate <120 beats/min) (*1*).

Although the mechanism of relative bradycardia is not known, a hypothesis is that increased levels of inflammatory cytokines, such as interleukin-6, which was reported for patients with COVID-19, can increase vagal tone and decrease heart rate variability (*4*–*6*). Another hypothesis is that the toxic effect on the nervous system caused by SARS-CoV-2 (*7*) disturbs autonomic control of heart rate. **Angiotensin-converting enzyme 2,** which is the receptor for SARS-CoV-2, is known to be expressed on cardiac cells (*8*). Therefore, relative bradycardia might reflect a characteristic inflammatory response to COVID-19, directly or indirectly affecting cardiovascular system.

There are several limitations in our study. First, 34 patients received antipyretic medicines during their hospitalization (acetaminophen, n = 33; loxoprofen, n = 1), and 1 patient received prednisolone (5 mg/day) for myasthenia gravis. Because fever was underestimated for patients who received these medications, relative bradycardia might be a more common clinical sign. In our cohort, body temperature decreased over time. Although there was a relationship between pulse rate and time after first symptom in a univariable model, this finding was probably confounded by body temperature and thus not significant when adjusted. Second, our data did not include patients who were intubated. Additional research on patients with severe respiratory dysfunction is needed.

In summary, relative bradycardia was a characteristic clinical finding in patients who had mild-to-moderate COVID-19 in Japan. This clinical sign could help clinicians diagnose COVID-19.

Appendix Additional information on relative bradycardia in patients with mild-to-moderate coronavirus disease, Japan.
