# Supplementary material for: Relative Bradycardia in Patients with Mild-to-Moderate Coronavirus Disease, Japan
Source: Emerg Infect Dis. 2020 Oct;26(10):2504–6. doi: 10.3201/eid2610.202648 (PMC7510693; doi:10.3201/eid2610.202648)
Supplement: Appendix — Additional information on relative bradycardia in patients with mild-to-moderate coronavirus disease, Japan. [file 20-2648-Techapp-s1.pdf]

Article DOI: https://doi.org/eid2610.202648

Relative Bradycardia in Patients with Mild-to-Moderate Coronavirus Disease, Japan

Appendix

Appendix Table. Association between pulse rate and other characteristics for coronavirus disease patients, by 2-level mixed-effects linear regression, Japan

		Univariable		Multivariable*	
Characteristic	No. observations	Coefficient	p value	Coefficient	p value
Age, y	313	0.03 (–0.14 to 0.19)	0.76		
Sex	313				
F	82	–4.53 (–9.30 to –0.23)	0.06		
M	231	Referent			
Time from first symptoms, d	313	–0.74 (–1.00 to –0.47)	<0.001		
Body temperature, °C†	313	7.37 (5.92–8.82)	<0.001	7.37 (5.92–8.82)	<0.001
Systolic blood pressure, mm Hg	302	0.12 (0.02–0.22)	0.02		
Diastolic blood pressure, mm Hg	302	–0.02 (–0.15 to 0.11)	0.76		
Respiratory rate/min	308	1.40 (0.76–2.04)	<0.001		
SpO2, %‡	313	–0.83 (–1.68 to 0.03)	0.06		

*p value for random intercept: <0.001 for all models. Variables without values were not included in the model.
†Only body temperature is significant.
‡SpO2, percutaneous oxygen saturation.
